# A Retrospective Outcomes Study of 20 Sacroiliac Joint Fusion Patients

**DOI:** 10.7759/cureus.260

**Published:** 2015-04-01

**Authors:** Carter E Beck, Saskia Jacobson, Eamon Thomasson

**Affiliations:** 1 Montana Neuroscience Institute; 2 Biomedical Engineering, Columbia University

**Keywords:** bone morphogenic protein, sacroiliac joint pain, fusion

## Abstract

**Study Design:**

Retrospective case series.

**Purpose:**

To report a novel approach to open posterior sacroiliac (SI) joint arthrodesis using a threaded titanium cage containing rhBMP­-2.

**Materials & Methods:**

Twenty consecutive patients with a mean age of 57.7 years (range: 33­-84) underwent posterior SI joint fusion. Two closely related novel posterior oblique approaches were employed. Enrolled subjects included 17 females and three males. The mean follow­-up time for CT to assess fusion was 27 months (range: 17­-45 months). Insurance included a mixture of public and private payers. One of the patients (patient 19) was on worker’s compensation. During follow-­up, patients were assessed radiologically for radiographic bony union and asked to rate their satisfaction with the procedure. The Oswestry Disability Index (ODI) was applied on a one-time basis upon follow-up. All patients were diagnosed with sacroiliac joint pain based primarily on a positive response to fluoroscopically guided injections into the sacroiliac joint.

**Results:**

Out of 20 patients, 33 SI joints were considered symptomatic and operated, and 32/33 joints successfully fused radiographically (a 96.9% fusion success rate). The average procedure satisfaction rating (PSR) was 7.25 out of a maximum 10 (range 1­-10). Seventeen patients responded to post-surgery ­follow-up questions, and 13 patients (76%), indicated they would elect to have the surgery again as well as recommend it to others. Average estimated blood loss was less than 50 mL, and average length of stay was one day.

**Conclusions:**

Preliminary experiences with these novel posterior approaches to the SI joint described here seem to be safe and effective. The novel posterior approaches to the SI joint described here appear, preliminarily, to have many advantages over previously described procedures including markedly reduced surgical morbidity.

## Introduction

Low back pain is ubiquitous, with a lifetime prevalence of 60­-80% [1-3]. It is often considered idiopathic, but a specific pain generator can actually be identified in approximately 75% of chronic cases [2]. The sacroiliac (SI) joint is an often overlooked source but is estimated to account for 16-­30% of patients diagnosed with low back pain [4-6]. Risk factors for SI joint dysfunction may include abnormal gait, scoliosis, arthritis, previous lumbar spinal surgery, trauma, and childbirth [5, 7]. Diagnosis remains problematic, with no universally accepted method [5]. Current best practice diagnostic techniques include pain provocation [2-3, 5, 8-10], diagnostic blocks [5, 10-11], and intra­articular fluoroscopically-guided injections [2, 7-8, 11]. Radiographic analysis has not proven to be sensitive or specific enough to be used alone, but it may be helpful when used in conjunction with other diagnostic techniques [11]. In the author’s experience, there are no reliable radiographic correlates for SI joint dysfunction.

The SI joint was initially suggested as a source of chronic low back pain in 1905 [5, 10, 12] but was eclipsed by the intervertebral disc as the main back pain generator studied in the 20th century. More recently, the sacroiliac joint is considered an important source of low back pain with a more comprehensive understanding of its etiology. Treatment for sacroiliac joint pain is often limited to non­operative, conservative care, including physical therapy, non­steroidal anti­inflammatory agents, intra­-articular injections, and radiofrequency neurotomy [2, 5, 8-11, 13]. Traditional SI joint fusion procedures are complex and invasive, involving open exposure of the joint with instrumented fixation and/or bone graft harvesting, and are typically associated with lengthy recovery times. Outcomes of traditional SI joint fusion procedures were observed to be so poor that these procedures were virtually abandoned over the last few decades. This retrospective study evaluates the safety and effectiveness of a novel arthrodesis technique using a minimally invasive approach with a single threaded fusion device inserted across the joint.

## Materials and methods

A comparative retrospective analysis was conducted on 20 consecutive patients who underwent sacroiliac joint fusion performed by a single neurosurgeon. After Institutional Review Board approval, patients were contacted for follow­-up and medical charts were systematically reviewed.

Mean age at the time of surgery was 57.7 years (range: 33-­84). There were 17 females and 3 males. Out of the 20 patients studied, 16 were non­smokers, two smoked more than 10 cigarettes a day upon follow-­up, and two were former smokers (Table 1). The mean follow-­up was 28.5 months (range: 17­-45 months), at which time patients were assessed radiologically via CT scan for bone union and asked to rate their experience with the SI joint fusion on a 1­-10 scale, with a score of 1 indicating the least satisfaction, and a score of 10 indicating the most satisfaction. The Oswestry Disability Index (ODI) was also applied on a one-time basis upon follow-up. In addition, patients were asked the following questions by phone after the follow­-up date: “In retrospect, would you have the surgery again?” and “Would you recommend the sacroiliac joint fusion surgery to others with similar lower back pain issues?” Of the twenty patients, two could not be reached to answer the postoperative follow-­up questions. The comorbidity of spinal surgery before, during, and after the study period was tabulated.

**Table 1:** Patient-Specific Details *M *male, *F* female

No.	Age	Sex	Smoker	Positive Joint Injections	Other Positive Criteria
20	75	F	No	Yes	Local tenderness
19	56	F	No	Yes	Local tenderness
18	59	F	No	Yes	Local tenderness
17	33	F	>10 cig/day	Yes	Local tenderness
16	62	F	No	Yes	Local tenderness
15	55	F	No	Yes	Local tenderness
14	52	F	No	Yes	Local tenderness
13	42	F	No	Yes	Local tenderness
12	42	F	No	Yes	None
11	49	F	Former	Yes	Local tenderness
10	64	F	No	Yes	Local tenderness
9	63	F	No	Yes	Local tenderness
8	71	M	Former	Yes	None
7	53	F	No	Yes	Local tenderness
6	52	F	No	Yes	Local tenderness
5	51	M	No	Yes	Local tenderness
4	79	F	No	Yes	Local tenderness
3	84	F	No	Yes	Local tenderness
2	60	M	No	Yes	Local tenderness
1	52	F	>10 cig/day	Yes	Local tenderness

Diagnostic criteria for this procedure included subjective reports of pain, which roughly equated to the SI joint region, positive point provocation, and localized pain in the SI joint. All of the patients tested positive on diagnostic/therapeutic intra­-articular sacroiliac injections using a local anesthetic and corticosteroid. Patients who reported substantial pain relief lasting one day or more following injection were deemed positive. CT and/or MRI imaging was used to examine the SI joint and exclude lumbar and hip pathology.

**Figure 1:**
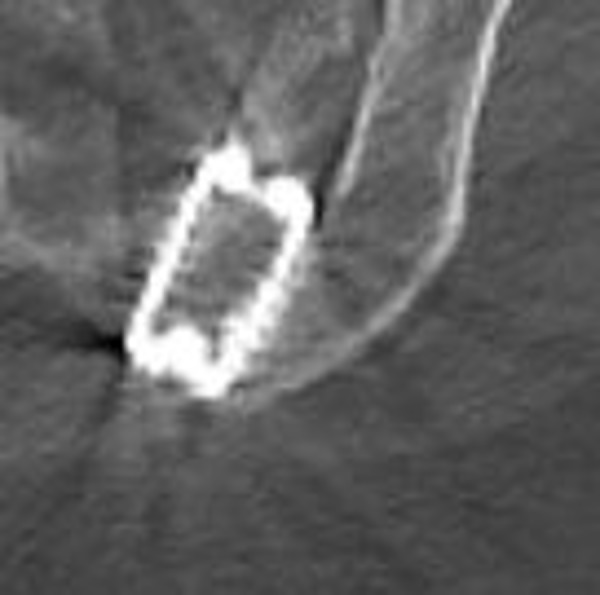
Axial CT of the left-sided posterior medial oblique fusion

**Figure 2:**
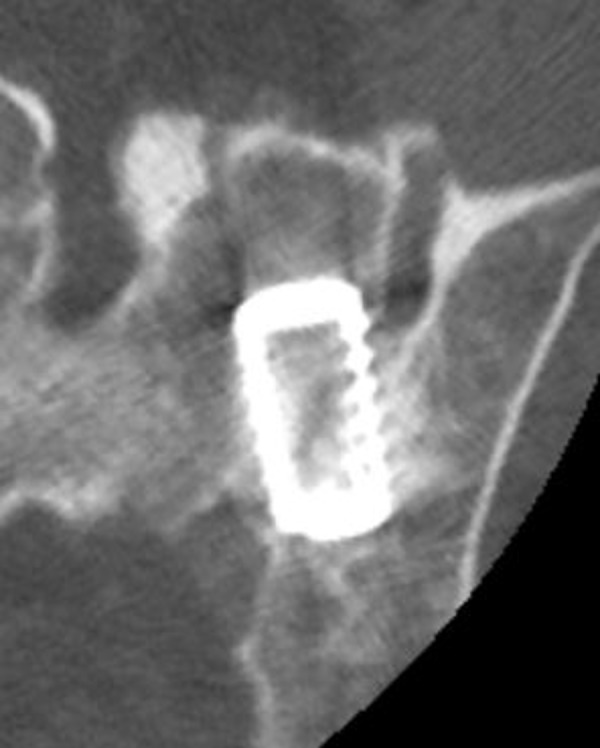
Axial CT of the left-sided posterior lateral oblique fusion

**Figure 3:**
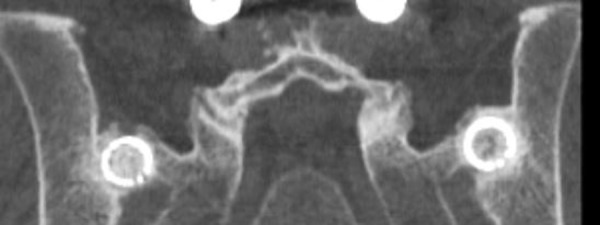
Coronal CT of bilateral posterior lateral oblique fusion

Patients were treated by two similar posterior mini open arthrodesis techniques using a single threaded titanium cage (INTERFIX, Medtronic, Memphis, TN) filled with INFUSE® (rhBMP-­2). Under general anesthesia, the patients were positioned prone on a laminectomy frame. A posterior medial oblique approach was initially employed to fixate the joint by driving a cage into the posterior ligamentous cleft between the sacrum and ilium (Figure 1). Due to ambiguities in the surgical anatomy, the procedure was modified into a more direct trans-cleft approach, which accomplished the same goal with greatly simplified surgical anatomy (Figures 2, 3). The latter procedure is described below. A 3 cm incision was made over the posterior superior iliac spine (PSIS). The overlying fatty tissues were divided, then the overlying fascia was divided, and a subperiosteal technique was employed to expose the PSIS. A working channel was then positioned over the PSIS, and the channel was angled approximately perpendicular to the floor in the rostral/caudal plane and at approximately 15­-30 degrees lateral to medial, depending on patient anatomy. A hand drill was used to cut a core 45­-55 mm deep through the ilium and sacral ala, across to the ligamentous cleft of the SI joint. A rhBMP­2 filled titanium cage was then threaded into the newly created channel so as to span the posterior ligamentous portion of the joint. Iliac bone bleeding was controlled using gelfoam. The fascia was sutured over the PSIS, and the sub­cutaneous tissues were closed with interrupted absorbable suture.

**Figure 4:**
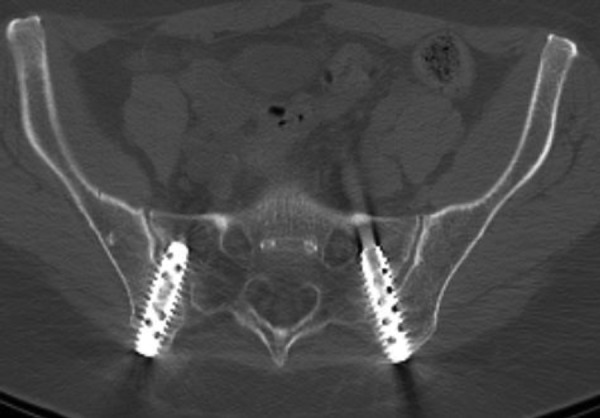
Axial CT showing trajectory of procedure

The first six patients underwent a posterior medial oblique procedure (Figure 1), whereas the remaining fourteen patients underwent a modified posterior lateral procedure (Figure 2). Thirteen patients underwent bilateral fusion procedures (Figures 3, 4) in which a cage was inserted into both sacroiliac joints, while six patients had a right side only fusion procedure, and one patient had a left side only fusion procedure. In the case of the bilateral procedure, one patient had operations for either side performed on different dates. A view of both joints showing the trajectory of the fusion procedures is shown in Figure 4. The raw procedural data is presented in Table 2.

**Table 2:** Procedural Details and Results by Patient *PSR* Procedure Satisfaction Rating, *M* male, *F* female, *dd* different dates

No.	Age	Sex	ODI Score	PSR	Type of Fusion	Fusion Success	Follow-up (Months)	Complications	Procedure Type
20	75	F	4	10	Bilateral	Yes	45	None	Medial oblique
19	56	F	44	8	Bilateral	Yes	19	None	Lateral
18	59	F	6	10	Bilateral	Yes	18	None	Lateral
17	33	F	28	7	Right	Yes	19	None	Lateral
16	62	F	4	9	Right	Yes	20	None	Lateral
15	55	F	26	4	Bilateral	Yes	19	None	Lateral
14	52	F	36	1	Bilateral	Yes	19	None	Lateral
13	42	F	56	4	Bilateral, dd	Yes	19	None	Lateral
12	42	F	24	10	Bilateral	Yes	23	None	Lateral
11	49	F	64	7	Left	Yes	17	None	Lateral
10	64	F	6	9	Right	Yes	43	None	Medial oblique
9	63	F	32	4	Bilateral	Not left	27	Left cage over-penetrated	Lateral
8	71	M	16	9	Bilateral	Yes	35	None	Medial oblique
7	53	F	18	7	Right	Yes	28	None	Lateral
6	52	F	2	10	Bilateral	Yes	39	None	Medial oblique
5	51	M	30	7	Right	Yes	41	None	Medial oblique
4	79	F	36	9	Right	Yes	19	None	Lateral
3	84	F	42	10	Bilateral	Yes	42	None	Medial oblique
2	60	M	0	10	Bilateral	Yes	36	None	Lateral
1	52	F	50	2	Bilateral	Yes	42	None	Lateral

## Results

The average estimated blood loss in this approach was 50 mL or less. Average length of stay was 0.95 days (one patient stayed two days, two patients went home the same day as their surgery, and all other patients were released the day after their surgery). Fusion, defined as radiographic evidence of trabecular bone bridging, was present in 32 out of 33 joints, for a fusion success rate of 96.9%. One joint approached with the posterior lateral technique did not fuse as the cage was placed too far into the ala and lost connection with the ilium.

There were no other significant surgical complications. Specifically, there were no infections, no bleeding events, no re­operations, and no medical complications.

**Figure 5:**
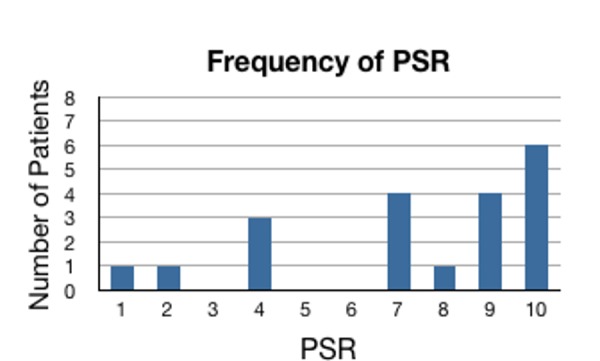
Frequency of PSR PSR: Procedure Satisfaction Rating

The average procedure satisfaction rating (PSR) (as measured on a sliding 1­-10 scale with 1 being the least satisfactory and 10 being the most satisfactory) was 7.25 (Figure 5). A rating of 1­-5 was considered unsatisfactory while a rating of 6­-10 was considered satisfactory. Six patients rated the procedure the highest possible value of 10 while 15 patients (75%) gave a score of 7 or higher. This left only five patients (25%) with a score of lower than 7. As can be seen in Table 2, the patients who gave unsatisfactory scores all received the posterolateral procedure.

Among the 18 patients (90%) who responded to the post-surgical follow-­up questions, 14 patients indicated they would elect to have the surgery again, and those same 14 patients responded that they would recommend the SI joint fusion procedure to others with similar low back pain issues. The remaining four patients who responded to the post-surgical follow-­up questions indicated they would not elect to have the surgery again, nor would they recommend the surgery to others.

The ODI was applied to all patients on a one-time basis upon follow-up to assess overall health perceptions of patients. The average ODI was a 26.2, which falls in the moderate disability category (21­-40). In total, one patient was scored in the crippling back pain category (61-­80), four patients were scored in the severe disability category (41-­60), seven patients were scored in the moderate disability category (21­-40), and eight patients were scored in the minimal disability category (0­-20).

**Figure 6:**
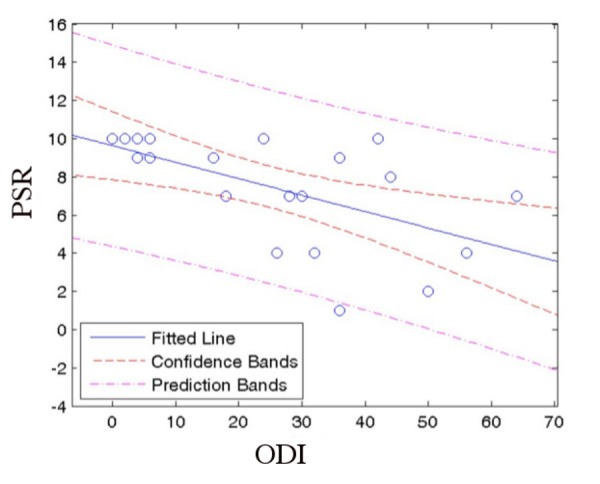
Linear regression fit of PSR vs. ODI with 95% confidence and prediction bands PSR: Procedure Satisfaction Rating ODI: Oswestry Disability Index

Figure 6 plots PSR versus ODI. The data indicates a correlation coefficient of ­-0.59 (P≤0.005). This is consistent with the ODI being high when the PSR is low in the majority of cases. The R^2^ value of 0.3465 indicates that 35% of the variation in PSR can be explained by the corresponding ODI, while the remaining 65% must be attributed to other factors. In several cases, a high PSR was obtained despite a high ODI.

**Figure 7:**
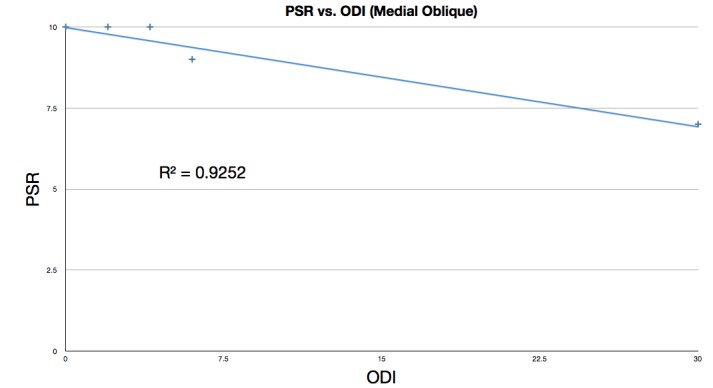
Linear regression fit of PSR vs. ODI for medial oblique procedure patients PSR: Procedure Satisfaction Rating ODI: Oswestry Disability Index

Figure 7 plots PSR versus ODI, including only the six patients who had a medial oblique procedure. The data yielded a correlation coefficient of -0.961. This is consistent with a high PSR corresponding with a low ODI. The R^2^ value of 0.9252 indicates that there is little variation from the trend.

**Figure 8:**
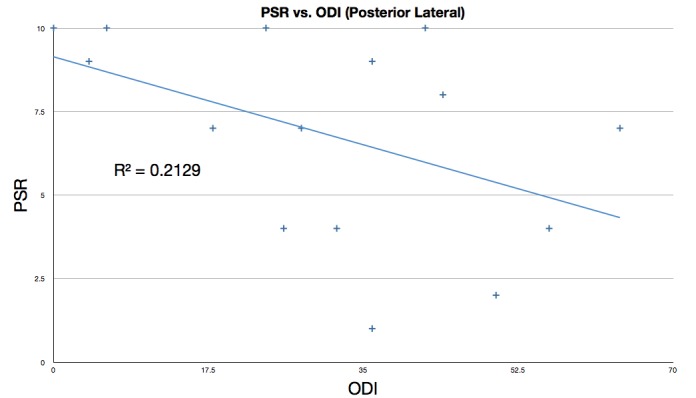
Linear regression fit of PSR vs. ODI for posterior lateral procedure patients PSR: Procedure Satisfaction Rating ODI: Oswestry Disability Index

Figure 8 plots PSR versus ODI, including the 14 patients who had the posterolateral procedure performed. The data yielded a correlation coefficient of -0.4614, showing only some correlation between high PSR and low ODI. The R^2^ value of 0.2129 shows that in this subset of patients, there is substantial variation from the trend of high PSR corresponding to low ODI.

## Discussion

The present case series reviews a single surgeon’s experience with novel posterior instrumented approaches to fixation of the SI joint. The results indicate surprisingly high patient satisfaction scores (Table 2). These procedures represent a substantial modification of earlier open posterior procedures wherein a portion of the ilium was translated into the sacral ala. The first cases were done with a posterior medial approach where the cage was essentially screwed into the ligamentous cleft between the sacral ala and the ilium (Figure 1). While apparently effective, the surgical anatomy proved to be ambiguous, variable, and at times confusing. The technique was modified into a more direct, more clearly defined and repeatable posterior lateral oblique procedure. This approach proved to be much more elegant. A similar anatomic result, fixation of the posterior cleft, is obtained (Figures 2, 3).

Posterior approaches offer direct access to fixation of the SI joint without significant surgical morbidity. The posterior superior iliac spine provides a bony entry point whose exposure requires little dissection, the transit of no functionally significant structure and is relatively superficial even in obese patients. Virtually all muscle trauma is thus obviated. Additionally, the trajectory of the fixation device poses little risk to neighboring neural and vascular structures. When considering elective surgical procedures for pain, in what may be a relatively pain sensitive patient population, minimized surgical trauma and complication should be of paramount importance. This point is underscored by the result shown here, where in this very early experience of 20 consecutive patients, there were no surgical complications.

Previous surgical approaches to SI joint fixation have met with such limited clinical success that they were essentially abandoned. Multiple explanations can be offered for this failure. Accurate diagnosis may be difficult due to the apparent absence of radiographic correlates of the pain syndrome. That is, as always, patient selection is likely very important for the successful surgical treatment of SI joint disease. These historical clinical failures of SI joint fixation procedures may in part be related to excessively traumatic surgical approaches. The relatively high patient satisfaction ratings presented here are likely due in part to the minimal surgical morbidity of the posterior instrumented approaches. There may also be a biomechanical explanation for what appear initially to be substantially improved results.

The normal healthy SI joint is thought to move very little or not at all, except around the time of childbirth in women. It is likely that in painful joints some sort of ligamentous laxity could allow for an abnormal micromotion. It is also likely that the instantaneous axis of rotation (IAR) for this motion exists relatively anterior within the true synovial portion of the joint. Procedures which attempt to fixate this large joint at or near the IAR are likely at a relative mechanical disadvantage to those, such as described here, which block the motion from some distance from the IAR.

The data presented in Figure 6 demonstrates that the correlation may be skewed because of medical and psychiatric factors not linked to the SI joint fusion that may confound patients’ abilities to interpret the results of surgery. Figure 8, which shows only the patients who received the posterior lateral procedure, shows greater dispersion than the medial oblique procedure. Two interpretations of the correlation between low ODI and high PSR are as follows: (1) Patients who started out with a lower ODI (less back pain) before the SI joint fusion procedure may have accorded the procedure a higher PSR than those patients with a higher ODI (with considerably more back pain); (2) The procedure resulted in significant improvement for the patients who gave it a higher PSR, even if they still scored a high postoperative ODI and thus still suffered substantial back pain.

The Oswestry Disability Index (ODI) data presented here is of limited value given that preoperative scales were not administered. However, even though preoperative ODI data is missing, it is still productive to examine the correlation between postoperative ODI and PSR. As can be seen in Figures 6-8, the data do show a positive correlation between the PSR for the procedure and a lower ODI. The data (particularly in Figure 8) also demonstrate very significant dispersion suggesting that the patient population has multiple co­morbidities which are substantially affecting their ODI. This idea is supported by the high incidence of surgical spinal disease in these patients (Table 3). A failure analysis of the five patients who gave the SI fusion procedure a PSR of lower than 7 also supports this notion. Patient 1, (PSR of 2), suffers from severe rheumatoid arthritis, chronic low back pain, and uses narcotics daily. Patient 9 (PSR 4) reported postoperatively that she was 90% better than she was prior to the surgery. Patient 11 (PSR 7) had the highest ODI, 6, indicating satisfaction with the procedure but poor overall health, further demonstrating the lack of consistency between ODI results and PSR.

**Table 3:** Pre- and Post-SI Joint Fusion Back Surgeries by Patient

No.	Pre SI Joint Fusion Back Surgery	Post SI Joint Fusion Back Surgery
20	Lumbar spondylostenosis with facet syndrome	None
19	None	None
18	None	L4-5 microlaminotomy & partial menial facetectomy
17	None	None
16	None	Right total hip arthroplasty
15	None	None
14	Fusion L5-S1	None
13	None	None
12	Fusion L5-S1	MN
11	Fusion C6-C7	None
10	None	None
9	None	None
8	Fusion L4-5	None
7	None	None
6	None	None
5	Thoracolumbar burst T12 laminectomy with posterolateral fusion from T11-L1	None
4	PLIF L1-2, L2-3, L3-4, L5	None
3	PLIF L3-4	None
2	L4-L5 S1, laminectomy, C4-5-6 fusion	L3-L4 fusion
1	Fusion L1-4	Fractured lumbar & fix, sciatic fusion

Patient 14 gave a PSR of 1, the lowest possible score. The patient underwent successful bilateral SI joint fusion but reported no relief of symptoms. The clinic notes indicate that failure was expected given that she had two SI joint injections which failed to provide much relief of her pain. The physician suggested surgery as a possible but unlikely solution to this patient’s moderate back pain (ODI of 36).

Patient 15 (PSR of 4), underwent successful bilateral fusion, but upon follow-up, the physician noted that she suffered from residual symptoms (aching over the L3 dermatome and numbness in her right foot) which were likely spinal in origin.

Three demographic features are noted to potentially skew the results of this study positively. First, the patients in this study were mostly non­smokers (16/20 or 80% never smoked while 2/20 had quit smoking). Furthermore, only one patient (5% of patients) was on worker’s compensation. One demographic feature which may skew the results in a negative way is that this study involved the investigator’s very early experience with a new procedure.

Several limitations to this study exist. First, the size of the cohort is relatively small. Second, this report describes a surgeon’s preliminary experience with a novel surgical technique using an off-the-shelf, non­optimized implant. Next, the ODI would have been more effective had it been applied preoperatively in addition to postoperatively in order to more effectively demonstrate changes in patients’ low back pain conditions.

## Conclusions

The present report describes a novel surgical technique with surprisingly good clinical results for a pain syndrome which has historically had an unsatisfactory response to surgical treatment. The posterior oblique surgical approaches represent a very promising avenue for treatment of sacroiliac-related pain syndromes.
